# High ROR1 Expression Is Associated With Poor Differentiation and Perineural Invasion in Cutaneous Squamous Cell Carcinoma

**DOI:** 10.1111/exd.70185

**Published:** 2025-12-07

**Authors:** Yannick Foerster, Kristine E. Mayer, Tilo Biedermann, Oana‐Diana Persa

**Affiliations:** ^1^ Department of Dermatology, TUM School of Medicine and Health Technical University of Munich Munich Germany; ^2^ Department of Dermatology University Hospital Cologne and Medical Faculty Cologne Germany

**Keywords:** cutaneous squamous cell carcinoma, ROR1, skin cancer, tyrosine kinase

## Abstract

Cutaneous squamous cell carcinoma (cSCC) is a common type of skin cancer, predominantly affecting elderly and immunosuppressed patients. Despite recent therapeutic advances, including the introduction of immune checkpoint inhibitors, outcomes for patients with metastatic or recurrent disease remain poor, underscoring the need for new therapeutic approaches and reliable biomarkers to identify patients at high risk of progression. In this context, receptor tyrosine kinase‐like orphan receptor 1 (ROR1), previously associated with poor prognosis and targeted therapeutically in other malignancies, has not yet been investigated in cSCC. This study aimed to evaluate ROR1 expression in cSCC and investigate its potential role as a biomarker for tumour aggressiveness. ROR1 expression was analysed via immunofluorescence in a tissue microarray of 360 cSCC samples from a biobank cohort at the University Hospital Cologne. Fluorescence intensity was quantified and correlated with clinicopathologic features and patient outcomes. High ROR1 expression was detected in 42.5% of samples, predominantly localised at tumour invasive fronts. Elevated ROR1 levels were significantly associated with poor tumour differentiation (*p* < 0.001), lymph node metastasis (*p* = 0.007), and perineural invasion (*p* = 0.005). Although higher ROR1 expression correlated with worse progression‐free and metastasis‐free survival, these differences did not reach statistical significance. In conclusion, this study identifies ROR1 as a novel marker of aggressive cSCC, linked to poor differentiation, lymphatic spread and perineural invasion. ROR1 holds potential both as a prognostic biomarker and as a therapeutic target, encouraging future exploration of ROR1‐directed therapies in advanced cSCC.

## 1 Introduction

Cutaneous squamous cell carcinoma (cSCC) is a common form of keratinocyte cancer (KC) [1, 2]. It affects primarily elderly patients with life‐long chronic sun exposure or under immunosuppression [1, 2, 3]. Metastatic disease is more frequent in men and people over the age of 75 [4]. Risk factors for local recurrence and metastasis include poor differentiation, perineural invasion, diameter > 2 cm, invasion beyond subcutaneous fat and localization on the lip, ear or temple [5]. Even though numerically more patients die because of tumour growth by local infiltration [6], distant metastasis is also associated with an extremely high mortality [7, 8, 9, 10]. Spread to distant organs often affects the lung and the axial skeleton among other organs [11].

The introduction of immune‐checkpoint inhibitors (ICI) has enlarged the set of promising therapeutic options, which had predominantly consisted of surgical excision and radiation therapy before [12, 13, 14]. Today, antibodies directed against programmed cell death protein 1 (anti‐PD‐1) are the first‐choice treatment option in patients with metastatic or locally advanced cSCC [13, 14, 15]. A meta‐analysis from 2022 describes a pooled objective response rate of 44% and a disease control rate of 66% [16]. As a second‐line option cetuximab, an antibody directed against epidermal growth factor receptor (EGFR), and/or chemotherapy is another systemic treatment option [13, 17, 18, 19]. EGFR is a tyrosine kinase receptor (RTK) which controls several downstream pathways including the MAPK/ERK and the PI3K/AKT/mTOR pathways which regulate cell maturation, proliferation and inhibition of apoptosis [20]. It was described that EGFR is overexpressed in about one third of cSCC cases and is associated with poor outcome [21]. Despite high overall response rates upon cetuximab immediately after ICI failure or as a first‐line treatment, reported median overall survival after cetuximab or chemotherapy treatment is less than 2 years [17, 22, 23]. For this reason, new therapies for advanced SCC are urgently needed.

In various hematologic malignancies the receptor tyrosine kinase‐like orphan receptor 1 (ROR1) has been described to be overexpressed and targeting of ROR1 is a promising treatment option currently tested in clinical trials [24, 25, 26]. The ligand for ROR1 is Wnt5a, which is expressed during embryonic development [27]. In adult tissue, however, mainly aberrant expression in various cancer types is reported. Besides lymphoma and leukaemia, ROR1 is also expressed in a set of solid malignancies like ovarian, breast, colon, lung, pancreatic and bladder cancer [28, 29, 30, 31, 32]. In most of the cancer types, ROR1 expression correlates with enhanced tumour growth and thus poor clinical outcome [32, 33, 34]. Furthermore, its expression correlates with epithelial‐mesenchymal transition (EMT) which typically occurs during cancer metastasis formation [34, 35]. As expression of ROR1 usually occurs in cancer tissue, but not in healthy tissue, this makes ROR1a promising therapeutic target. Different treatment modalities including small molecule inhibitors, antibody‐based therapy and even CAR T‐cell therapy, have lately been proposed to target ROR1‐expressing tumour cells [24, 25, 26, 36, 37, 38]. These interesting properties raise the question of whether ROR1 may also play a role in KC. However, ROR1 expression has not yet been investigated in cSCC.

So far, all cSCC patients receive similar follow‐up independently of their respective tumour cell biology. Even though clinical risk factors like tumour thickness and localization of the tumour are established, further biomarkers considering tumour biology may help to identify the patients requiring close follow‐ups because of a high risk for local recurrence or metastasis formation. Moreover, the expression of ROR1 on cSCC would make patients eligible for therapies directed against ROR1 taking advantage of their development for other indications.

## 2 Material and Methods

### 2.1 Patient Samples

Our study included 360 patients with cSCC who were diagnosed at the Department of Dermatology, University Hospital Cologne, and were included in the biobank (Figure S1). The study was approved by the Ethics Committee of the University of Cologne, and all samples were reviewed by at least one board‐certified dermato‐pathologist. Informed consent was obtained from all participants. Available clinical patient data were collected from electronic medical records.

### 2.2 Tissue Microarray Preparation

Formalin‐fixed, paraffin‐embedded tissue samples were used for tissue microarray (TMA) preparation as previously described [39]. Haematoxylin and eosin (HE)‐stained slides were reviewed to identify representative tumour regions, and core tissue biopsies (2.0 mm in diameter) were extracted from selected tumour areas of donor blocks by using a manual tissue microarrayer (MTA‐1, AlphaMetrix Biotech, Rödermark, Germany) Each core was then transferred into a recipient paraffin block in a pre‐specified array format. After embedding, TMA blocks were sectioned at 5 μm thickness using a rotary microtome and mounted on glass slides. Quality control was performed by examining HE‐stained sections to confirm accurate tissue placement and preservation.

### 2.3 Immunofluorescence Staining

Paraffin‐embedded TMA slides were deparaffinised by immersion in xylene, followed by isopropanol and a graded ethanol series (100%, 96%, 75%), and then rehydrated in distilled water. Antigen retrieval was performed using the 2100 Retriever pressure cooker (Aptum Biologics Ltd., Southampton, UK) for optimal epitope exposure. Sections were then blocked with 10% goat serum for 30 min to minimise nonspecific binding and incubated overnight at 4°C with the anti‐ROR1 primary antibody (Dilution 1/200, PA5‐96241, Thermo Fisher Scientific, Waltham, MA, USA), followed by fluorophore‐conjugated goat anti‐rabbit IgG antibody (Dilution 1/400, A11012, Thermo Fisher Scientific) for 1 h at room temperature. Nuclei were counterstained with DAPI (Dilution 1/1000), and slides were mounted using Mowiol 4–88 (81 381, Sigma, Burlington, MA, USA). All slides were stained simultaneously to maintain comparability.

### 2.4 Imaging and Statistics

Fluorescence imaging was performed using the Keyence BZ‐X800 fluorescence microscope (Osaka, Japan). Samples were visualised and captured at a magnification of 1000×, and images were acquired under standardised exposure settings to ensure consistency across samples. All images were processed and stitched together using the Keyence BZ‐X Analyzer software. Mean grey values (MGV) were measured to quantify fluorescence intensity using ImageJ 1.54 (National Institutes of Health, Bethesda, MD, USA), and values were standardised to the overall mean. Samples were then divided based on the standardised MGV (immunoreactivity, IR): ROR1 expression was classified as low for MGV ≤ 1 and high for MGV > 1. All statistical analyses were performed with R studio 4.1.1 (Posit PBC, Boston, MA, USA). Non‐parametric tests were applied for data analysis, including the Wilcoxon‐Mann–Whitney test for pairwise comparisons and the Kruskal‐Wallis test for group comparisons. Chi [2], test (sample size > 5) and Fisher's exact test (sample size ≤ 5) were used to assess associations between categorical variables. Statistical significance was defined as *p* < 0.05 (*p* < 0.05*, *p* < 0.01**, *p* < 0.001***). No imputations of missing values were made.

## 3 Results

### 3.1 Patient Population

ROR1 expression was evaluated in a total of 360 cSCC samples (Table 1). A total of 266 patients (73.9%) were male, and 94 (26.1%) were female. The mean age at diagnosis was 78.41 years (standard deviation (SD): 10.31 years). The most common tumour site was the scalp (215 cases, 59.7%), followed by the lower extremity (37 cases, 10.3%), ear (28 cases, 7.8%), upper extremity (26 cases, 7.2%), nose (24 cases, 6.7%), trunk (17 cases, 4.7%), and lip (13 cases, 3.6%). The mean tumour thickness was 4.04 ± 3.39 mm, and the mean tumour diameter was 2.05 ± 2.30 cm. Most tumours were well‐differentiated (G1, 236 cases, 65.6%) or moderately differentiated (G2, 68 cases, 18.9%). Poorly differentiated (G3, 39 cases, 10.8%) and undifferentiated tumours (G4, 17 cases, 4.7%) were less common. Perineural invasion was present in 12 tumour samples (3.3%). Follow‐up was available for 238 patients. The median overall survival (OS) was 127.6 months, with a median recurrence‐free survival (LRFS) of 43.28 months. Median progression‐free survival (PFS) and metastasis‐free survival (MFS) were 39.13 months and 42.06 months, respectively.

**TABLE 1:** Baseline patient data. A total of 360 patients were included in the study. ROR1 expression was assessed based on standardised mean grey value (MGV) of immunoreactivity (IR) and classified as low (MGV ≤ 1) or high (MGV > 1). SD, standard deviation; 95% CI, 95% confidence interval.

Patient data	Total [%]	ROR1high [%]	ROR1low [%]	ROR1 IR
Gender	360 [100]	153 [100]	207 [100]	
Male	266 [73.9]	112 [73.2]	154 [74.4]	1.00 ± 0.57
Female	94 [26.1]	41 [26.8]	53 [25.6]	0.99 ± 0.53
Age (years)				
Mean ± SD	78.41 ± 10.31	78.09 ± 10.96	78.64 ± 9.83	
Localisation	360 [100]	153 [100]	207 [100]	
Scalp	215 [59.7]	86 [56.2]	129 [62.3]	0.98 ± 0.60
Lip	13 [3.6]	5 [3.3]	8 [3.9]	0.97 ± 0.58
Ear	28 [7.8]	13 [8.5]	15 [7.2]	1.07 ± 0.57
Trunk	17 [4.7]	6 [3.9]	11 [5.3]	1.01 ± 0.58
Upper extremity	26 [7.2]	10 [6.5]	16 [7.7]	0.98 ± 0.36
Lower extremity	37 [10.3]	22 [14.4]	15 [7.2]	1.10 ± 0.51
Nose	24 [6.7]	11 [7.2]	13 [6.3]	0.96 ± 0.47
Tumour thickness [mm]				
Mean ± SD	4.04 ± 3.39	3.95 ± 3.50	4.11 ± 3.32	
Tumour diameter [cm]				
Mean ± SD	2.05 ± 2.30	2.22 ± 3.12	1.92 ± 1.43	
Metastasis	360 [100]	153 [100]	207 [100]	
Lymph node	27 [7.5]	17 [11.1]	10 [4.8]	1.21 ± 0.67
Skin	6 [1.7]	0 [0]	6 [2.9]	0.49 ± 0.26
No metastasis	327 [90.8]	136 [88.9]	191 [92.3]	0.99 ± 0.55
G status	360 [100]	153 [100]	207 [100]	
1 (well)	236 [65.6]	83 [54.2]	153 [73.9]	0.90 ± 0.47
2 (moderate)	68 [18.9]	25 [16.3]	43 [20.8]	0.89 ± 0.48
3 (poor)	39 [10.8]	31 [20.3]	8 [3.9]	1.56 ± 0.64
4 (undifferentiated)	17 [4.7]	14 [9.2]	3 [1.4]	1.61 ± 0.72
Perineural invasion	360 [100]	153 [100]	207 [100]	
No	348 [96.7]	143 [93.5]	205 [99.0]	0.98 ± 0.55
Yes	12 [3.3]	10 [6.5]	2 [1.0]	1.48 ± 0.74
Desmoplasia	360 [100]	153 [100]	207 [100]	
No	348 [96.7]	145 [94.8]	203 [98.1]	0.99 ± 0.56
Yes	12 [3.3]	8 [5.2]	4 [1.9]	1.25 ± 0.50
Relapse	356 [100]	153 [100]	203 [100]	
Yes	20 [5.6]	8 [5.2]	12 [5.9]	1.01 ± 0.72
No	336 [94.4]	145 [94.8]	191 [94.1]	1.00 ± 0.55
Immunosuppression	359 [100]	152 [100]	207 [100]	
Yes	58 [16.2]	25 [16.4]	33 [15.9]	0.99 ± 0.58
No	301 [83.8]	127 [83.6]	174 [84.1]	1.00 ± 0.56
Hematologic malignancy	360 [100]	153 [100]	207 [100]	
Yes	33 [9.2]	16 [10.5]	17 [8.2]	1.09 ± 0.60
No	327 [90.8]	137 [89.5]	190 [91.2]	0.99 ± 0.56
Tumour proportion score (number of PD‐L1‐positive tumour cells/total number of tumour cells) x 100				
Mean ± SD	19.15 ± 33.83	18.57 ± 33.54	19.58 ± 34.13	
Overall survival (OS) [months]				
Median [95% CI]	127.60 [121.58; 133.62]	130.13 [123.55; 136.71]	48.19 [45.78; 50.61]	
Recurrence‐free survival (LRFS) [months]				
Median [95% CI]	43.28 [41.33; 45.22]	43.96 [41.32; 46.61]	42.61 [39.77; 45.45]	
Progression‐free survival (PFS) [months]				
Median [95% CI]	39.13 [36.62; 41.64]	38.22 [34.49; 41.95]	40.12 [36.82; 43.41]	
Metastasis‐free survival (MFS) [months]				
Median [95% CI]	42.06 [39.55; 44.56]	41.10 [37.33; 44.86]	41.44 [38.36; 44.51]	

### 3.2 Undifferentiated and Poorly Differentiated Tumours Show High ROR1 Expression

**FIGURE 1:**
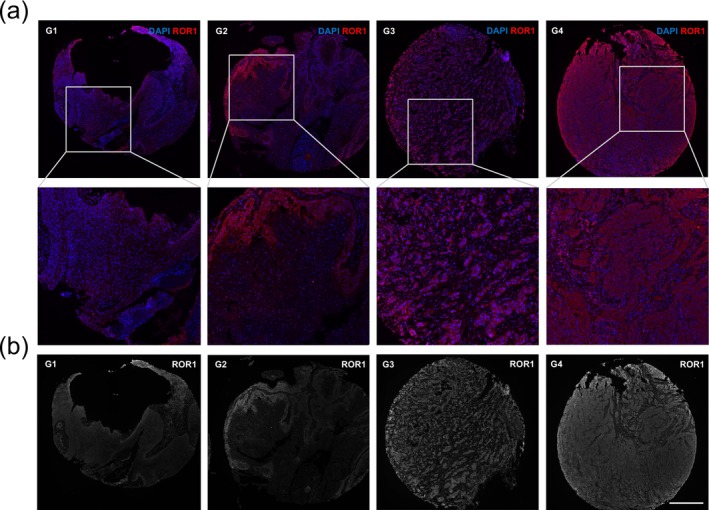
(a) Tissue microarrays (TMA) of cutaneous squamous cell carcinoma (cSCC). The expression of receptor tyrosine kinase‐like orphan receptor 1 (ROR1) gradually increases with tumour dedifferentiation. Well‐differentiated (G1) and moderately differentiated (G2) tumour samples exhibited lower levels of ROR1 compared to poorly differentiated (G3) and undifferentiated (G4) samples. ROR1 expression was predominantly localised at the tumour cell margins, corresponding to the invasive front. (b) ROR1 expression is shown in grey. Mean grey values (MGV) were measured to quantitatively analyse fluorescence staining intensity. Scale bar: 500 μm.

**FIGURE 2:**
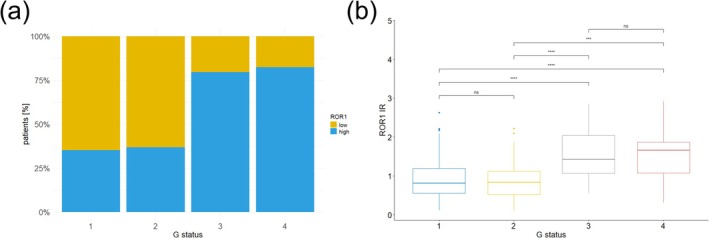
(a) Proportion [%] of patients with well‐differentiated (G1), moderately differentiated (G2), poorly differentiated (G3) and undifferentiated (G4) tumours stratified by receptor tyrosine kinase‐like orphan receptor 1 (ROR1) expression levels. ROR1 expression was assessed based on standardised mean grey value (MGV) of immunoreactivity (IR) and classified as low (MGV ≤ 1) or high (MGV > 1). The majority of G1 (153/236 cases) and G2 tumours (43/68 cases) exhibited low ROR1 expression levels, whereas the majority of G3 (31/39 cases) and G4 (14/17) tumours showed high ROR1 expression levels. The difference in distribution between groups was statistically significant (*p* < 0.001). (b) ROR1 expression levels were significantly lower in G1/G2 primary tumours compared to G3/G4 tumours (*p* < 0.001). No significant differences were detected between G1 and G2 tumours or between G3 and G4 tumours. (*p* < 0.05*, *p* < 0.01**, p < 0.001***).

Samples were categorised by standardised MGV (IR), with ROR1 expression classified as low (MGV ≤ 1) or high (MGV > 1) (Table 1). High ROR1 expression was observed in 153 cases (42.5%), while 207 cases (57.5%) showed low expression. ROR1 expression was heterogeneously distributed across the tumour samples, with high expression observed at the margins of tumour nests, particularly at the invasive front (Figure 1A,B). High ROR1 expression was associated with poorly differentiated and undifferentiated tumours (*p* < 0.001) (Figure 2A). Standardised MGV (IR) scores for G1/G2 tumours were significantly lower than those for G3/G4 tumours (*p* < 0.001) (Figure 2B). No significant differences were observed between G1 and G2 tumours, nor between G3 and G4 tumours. No ROR1 expression was detected in keratinocytes of the healthy epidermis (Figure S2).

### 3.3 High ROR1 Expression Is Associated With Lymph Node Metastasis

**FIGURE 3:**
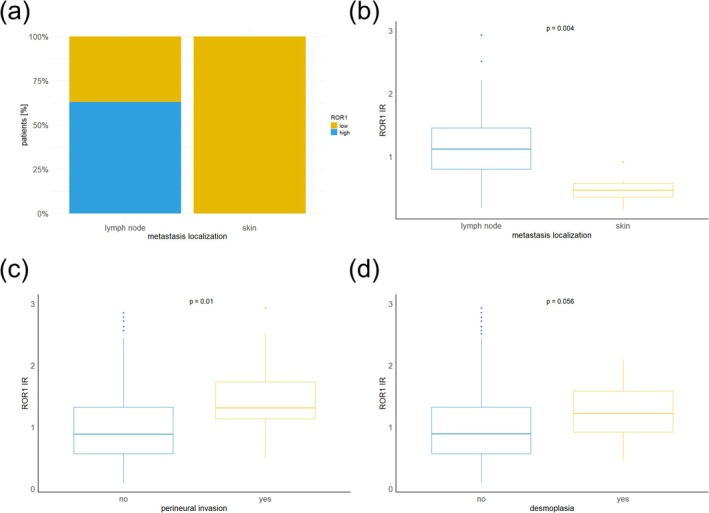
(a) Proportion [%] of patients presenting with lymph node or skin metastases stratified by receptor tyrosine kinase‐like orphan receptor 1 (ROR1) expression levels in their respective primary tumours. ROR1 expression was assessed based on standardised mean grey value (MGV) of immunoreactivity (IR) and classified as low (MGV ≤ 1) or high (MGV > 1). Among patients with lymph node metastases, the majority (17 of 27 cases) exhibited high ROR1 expression in the primary tumour. In contrast, all patients with skin metastases (*n* = 6) demonstrated low ROR1 expression. The difference in distribution between groups was statistically significant (*p* = 0.007). (b) Patients with lymph node involvement showed significantly higher levels of ROR1 in the primary tumour site compared to patients with skin metastases (*p* = 0.004). (c) Patients with perineural invasion showed significantly higher levels of ROR1 in the primary tumour site compared to those lacking perineural invasion (*p* = 0.01). (d) Patients with desmoplastic primary tumours showed higher levels of ROR1 compared to non‐desmoplastic tumours (*p* = 0.056).

All primary tumours from patients with skin metastases showed low ROR1 expression (6 cases) (Table 1). In contrast, the majority of tumours from patients with lymph node metastases (17/27 cases) exhibited high ROR1 expression (Figure 3A). When comparing patients with skin metastases to those with lymph node metastases, we observed a significantly higher frequency of tumours with high ROR1 expression among patients with lymph node involvement (*p = 0.007*). Also, tumour samples from patients with skin metastases showed significantly lower standardised MGV (IR) scores compared to patients with lymph node involvement (*p = 0.004*) (Figure 3B).

### 3.4 High ROR1 Expression Is Associated With Perineural Invasion

Of the 12 tumour samples with perineural invasion (3.3%), 10 exhibited high ROR1 expression, while only 2 samples with low ROR1 expression showed perineural invasion (*p* = 0.005) (Table 1). Consistently, tumour samples from patients with perineural invasion had significantly higher standardised MGV (IR) scores compared to those without perineural invasion (*p* = 0.01) (Figure 3C). The majority of patients with desmoplasia also had high ROR1 expression (8 cases) while only 4 patients with low ROR1 expression showed desmoplasia. However, this difference was not statistically significant (*p* = 0.078). Desmoplastic tumour samples also had higher standardised MGV (IR) scores compared to non‐desmoplastic tumour samples (*p* = 0.056) (Figure 3D).

### 3.5 G1/G2 Tumours Exhibit Better Progression‐Free Survival and Metastasis‐Free Survival

**FIGURE 4:**
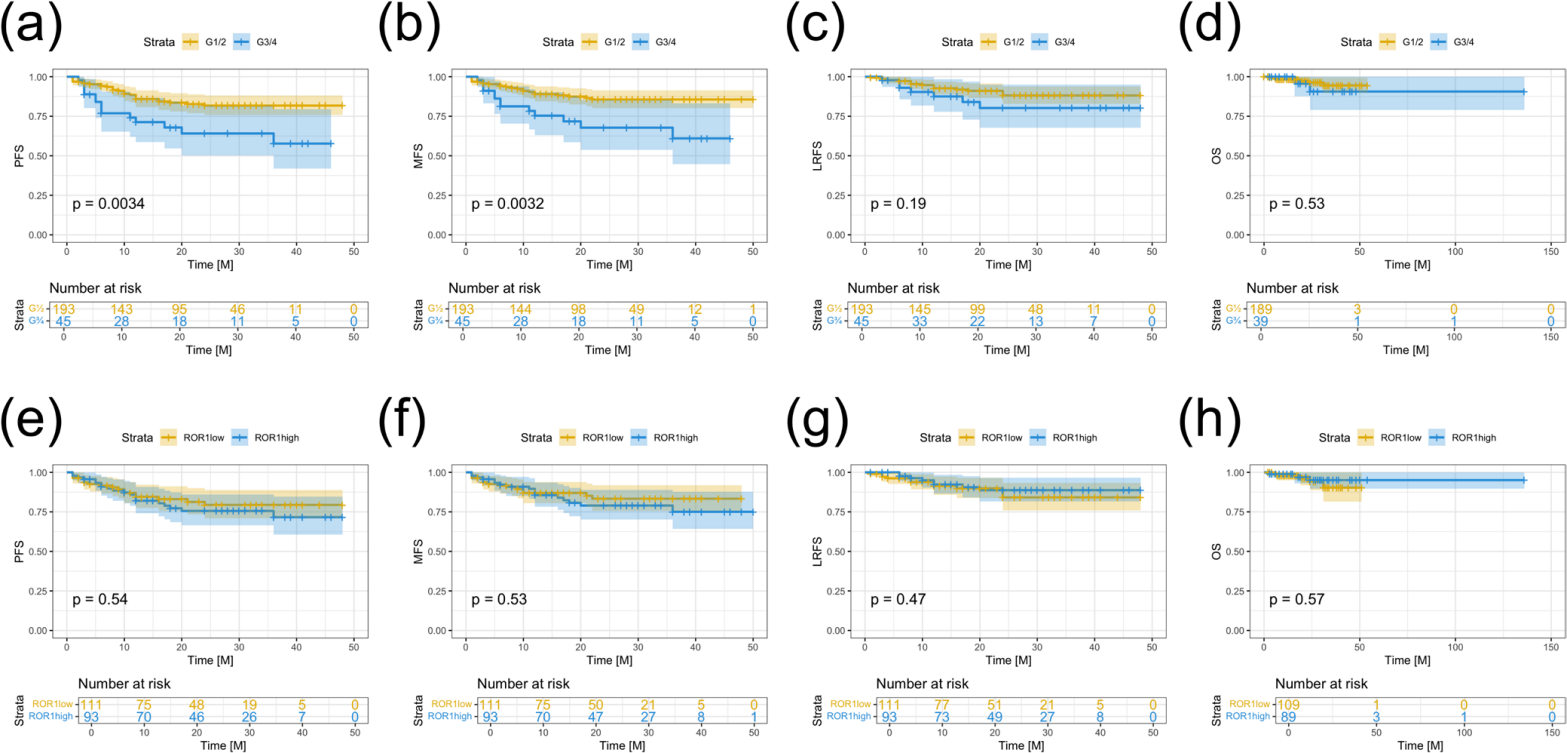
(a, b) Patients with well‐differentiated (G1) and moderately differentiated (G2) tumours exhibited significantly improved progression‐free survival (PFS) (*p* = 0.0034) and metastasis‐free survival (MFS) (*p* = 0.0032) compared to those with poorly differentiated (G3) or undifferentiated (G4) tumours. (c, d) No significant differences were observed between these groups in locoregional recurrence‐free survival (LRFS) or overall survival (OS). (e, f) Patients with low ROR1 expression showed higher absolute PFS and MFS compared to those with high ROR1 expression; however, these differences did not reach statistical significance. (g, h) Similarly, no significant differences in LRFS or OS were observed between patients with high versus low ROR1 expression.

In our cohort, patients with well‐differentiated (G1) and moderately differentiated (G2) tumours demonstrated significantly better progression‐free survival (PFS) (*p* = 0.0034) (Figure 4A), and metastasis‐free survival (MFS) (*p* = 0.0032) (Figure 4B), compared to those with poorly differentiated (G3) or undifferentiated (G4) tumours. There were no significant differences between these groups in locoregional recurrence‐free survival (LRFS) or overall survival (OS) (Figure 4C,D). Although patients with low ROR1 expression had higher PFS (Figure 4E), and MFS (Figure 4F), than those with high ROR1 expression, these differences were not statistically significant. Similarly, no significant differences were observed in LRFS or OS between patients with high and low ROR1 expression (Figure 4G,H).

### 3.6 ROR1 Expression in Immunocompromised and Patients With Hematologic Malignancies

Immunosuppression was present in 58 of 359 patients (16.2%), with similar proportions in the ROR1‐high (25/152; 16.4%) and ROR1‐low groups (33/207; 15.9%) (Table 1). ROR1 IR did not differ between immunosuppressed and non‐immunosuppressed patients. A history of hematologic malignancy was documented in 33 of 360 patients (9.2%). Sixteen of these patients (10.5%) were classified as ROR1 high and 17 (8.2%) as ROR1 low. ROR1 IR was slightly higher in patients with hematologic malignancies compared with those without (1.09 ± 0.60 vs. 0.99 ± 0.56). Differences in frequencies and ROR1 IR were not statistically significant.

## 4 Discussion

This study describes the expression of ROR1 in cSCC and characterises the potential of ROR1 as a biomarker and potential therapeutic target revealing three key findings. First, poorly differentiated and undifferentiated tumours show higher ROR1 expression. Second, high ROR1 expression in primary tumours is associated with lymph node metastasis formation. Finally, tumours with perineural invasion show higher ROR1 expression compared to tumours without this negative prognostic marker.

While ROR1 expression has been described in other tumour entities such as chronic lymphatic leukaemia, triple negative breast cancer and non‐small lung cancer, this is to our knowledge the first study which investigates ROR1 expression in cSCC. We analysed ROR1 expression on primary tumour slides from a representative cohort of cSCC which consisted of mainly male patients with a mean age at diagnosis of 78.41 years. Because immunosuppression and hematologic malignancies are established risk factors for cSCC and ROR1 is known to be overexpressed in several hematologic neoplasms, we additionally assessed whether ROR1 expression was enriched in patients with hematologic malignancies. Although these patients showed a slightly higher proportion of ROR1‐high tumours, the difference was not statistically significant. Higher ROR1 expression was found in poorly differentiated and undifferentiated tumours compared to tumours with a higher degree of differentiation. These findings are in line with data on lung carcinoma where high ROR1 expression was also associated with poor differentiation [40]. In various cancer types ROR1 expression has been described to stimulate EMT which facilitates metastasis formation [35]. Regarding metastasis formation, we found that most tumours from patients with lymph node metastases exhibited high ROR1 expression in the primary tumour. In contrast, ROR1 expression was not elevated in patients suffering from skin metastasis. Since ROR1 facilitates EMT, lymphatic metastasis formation seems to be more likely while skin metastasis formation may be driven by other mechanisms which do not seem to be affected by ROR1 expression. Similarly, Heabah and colleagues observed an association of ROR1 expression in lung carcinoma with a positive lymph nodal status [40].

Since ROR1 expression is connected to poorly differentiated tumours and tumours with lymphatic metastasis formation, it may be used as a diagnostic marker to evaluate the aggressiveness of the tumour. Other well‐described risk factors of cSCC are perineural invasion and desmoplasia. In this study, perineural invasion was associated with significantly higher expression of ROR1 compared to tumour samples without perineural invasion. Also, higher levels of ROR1 were detected in desmoplastic tumour samples compared to non‐desmoplastic tumour samples, even though statistically not significant. Through its association with poor differentiation, risk factors such as perineural invasion and desmoplasia, as well as metastasis formation, ROR1 may be viewed as a diagnostic marker for a higher aggressiveness of the tumour. Accordingly, the aftercare regimen may be adjusted in affected patients.

Poor differentiation of primary tumours was associated with significantly worse PFS and MFS compared with well‐ or moderately differentiated tumours. In contrast, LRFS and OS were not affected by tumour differentiation. Similarly, low ROR1 expression correlated with longer PFS and MFS, even though it was statistically not significant. One potential reason for this discrepancy is the fact that follow‐up was only available for 238 patients. While survival is not significantly influenced by ROR1 expression, it could still serve as a potential therapeutic target. Lately, ROR1 small molecule inhibitors, monoclonal antibodies and CAR T‐cells with ROR1 as target protein have been developed for a variety of different cancer entities [41, 42, 43, 44, 45]. Phase I studies have shown the safety of targeting ROR1 in patients with chronic lymphocytic leukaemia, triple‐negative breast cancer and non–small cell lung cancer [46, 47]. With a raising number of early clinical studies ongoing and in planning the evaluation of medicines directed against ROR1 may be transferred to KC and in particular metastasized cSCC where currently only limited treatment options are available.

In conclusion, this study evaluated ROR1 expression in a representative cohort of cSCC patients and revealed expression in the majority of tumour samples. High ROR1 expression was associated with poor differentiation, lymphatic metastasis formation and perineural invasion which may qualify ROR1 as a potential biomarker and therapeutic target structure in cSCC.

## Author Contributions

**Yannick Foerster:** data curation, writing original draft, conceptualization, methodology, formal analysis, visualisation. **Kristine E. Mayer:** data curation, methodology, writing original draft. **Tilo Biedermann:** supervision, writing review and editing, resources. **Oana‐Diana Persa:** supervision, validation, methodology, writing review and editing.

## Funding

The authors have nothing to report.

## Ethics Statement

The study was approved by the ethics committee of the University of Cologne.

## Conflicts of Interest

The authors declare no conflicts of interest.

## Supporting information


**Figure S1:** Flowchart of included tumour samples. A total of 445 samples were initially selected for tissue microarray (TMA) preparation. After removing duplicates, 420 patients were included in the biobank. Following the exclusion of samples lacking tumour tissue, a total of 360 samples were retained for analysis.

**Figure S2:** Tissue microarray (TMA) of cutaneous squamous cell carcinoma (cSCC) with adjacent epidermis. Scale bar: 500 μm.

## Data Availability

The data that support the findings of this study are available on request from the corresponding author. The data are not publicly available due to privacy or ethical restrictions.
