# Supplementary figures and images for: High ROR1 Expression Is Associated With Poor Differentiation and Perineural Invasion in Cutaneous Squamous Cell Carcinoma
Source: Exp Dermatol. 2025 Dec 7;34(12):e70185. doi: 10.1111/exd.70185 (PMC12683217; doi:10.1111/exd.70185)

445 total samples

Duplicates

420 samples in biobank

No tumor in TMA

360 samples analyzed

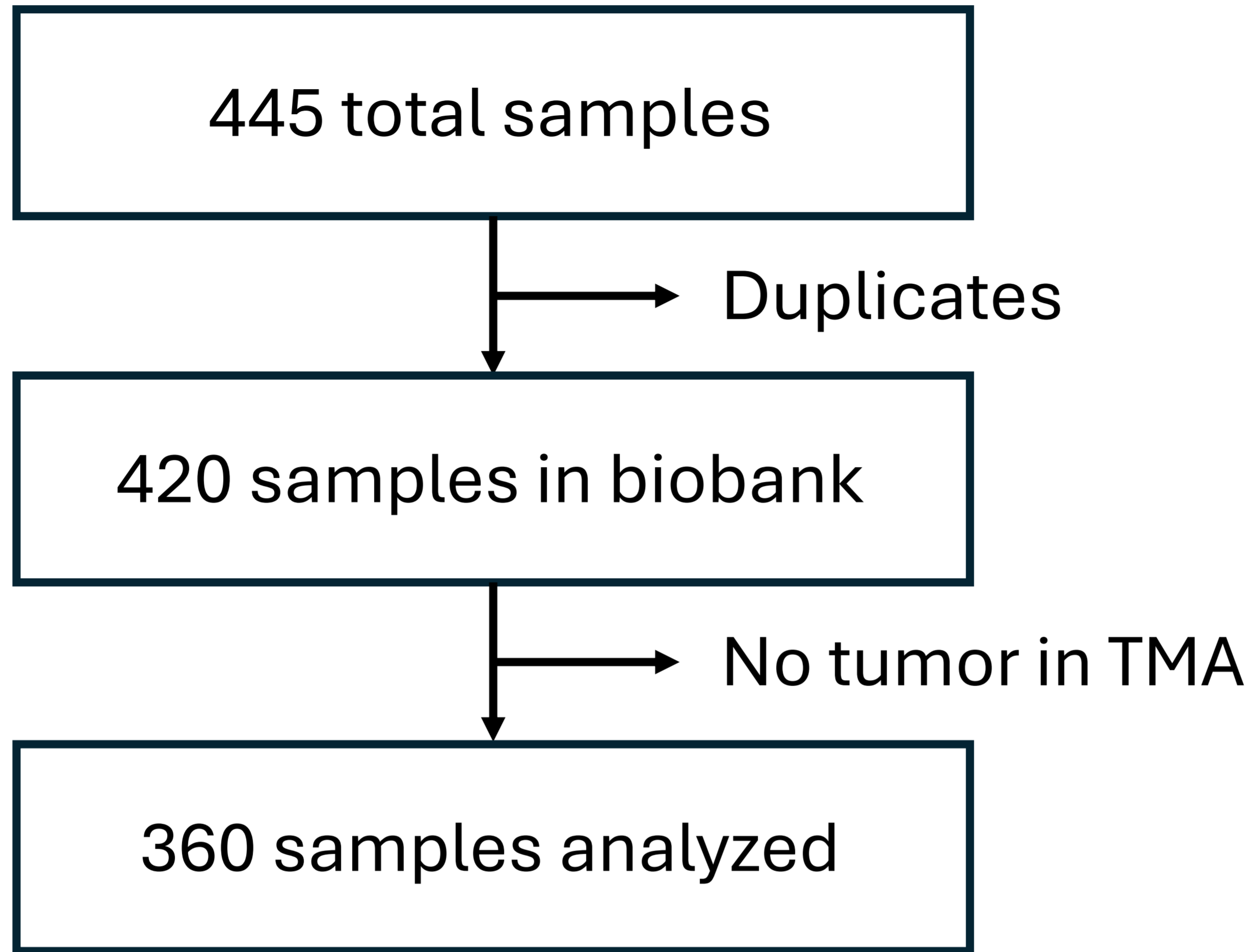

Supplement: Supplementary file 1 — Figure S1: Flowchart of included tumour samples. A total of 445 samples were initially selected for tissue microarray (TMA) preparation. After removing duplicates, 420 patients were included in the biobank. Following the exclusion of samples lacking tumour tissue, a total of 360 samples were retained for analysis. [file EXD-34-e70185-s002.pdf]

DAPI ROR1

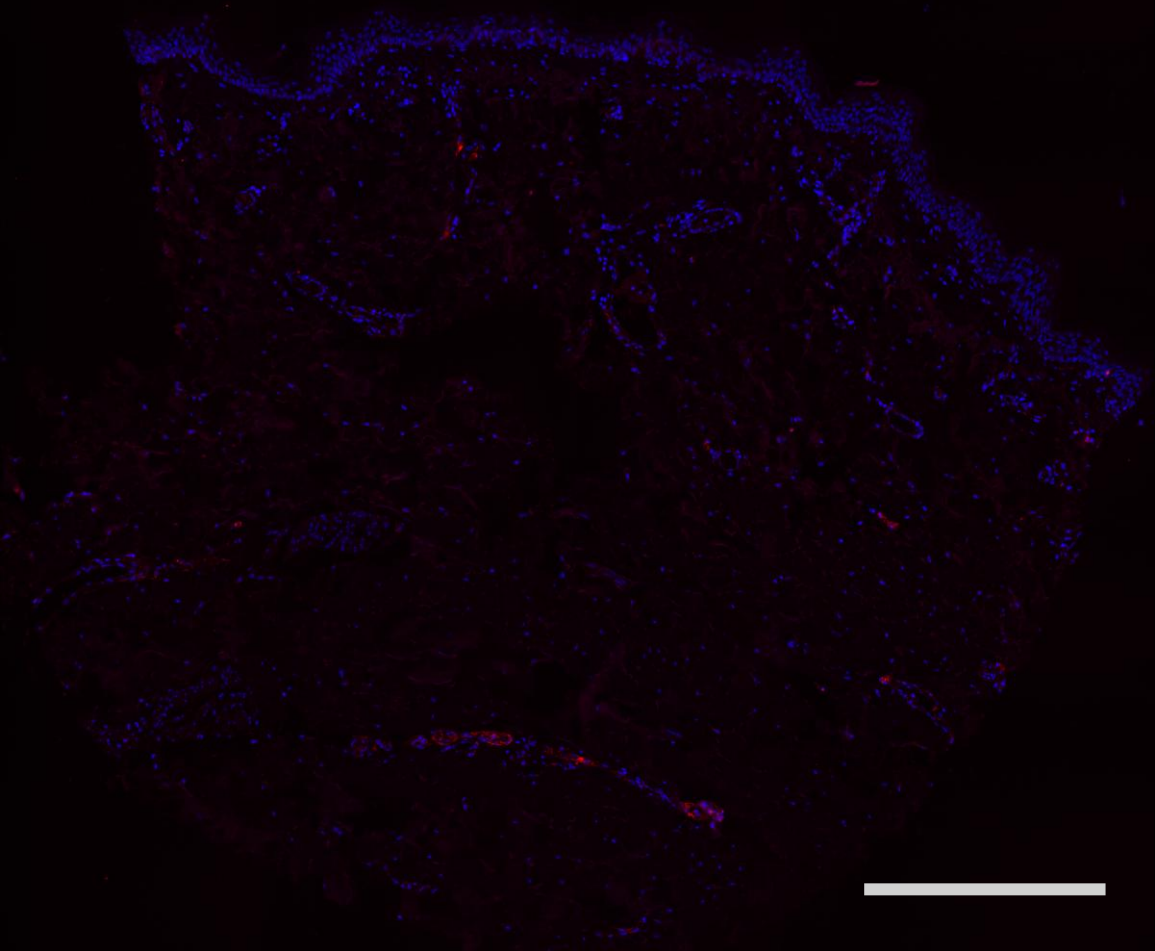

Supplement: Supplementary file 2 — Figure S2: Tissue microarray (TMA) of cutaneous squamous cell carcinoma (cSCC) with adjacent epidermis. Scale bar: 500 μm. [file EXD-34-e70185-s001.pdf]
